# Factors Affecting Treatment Adherence Among Persons With Multidrug-Resistant Tuberculosis (MDR-TB) in Western India: A Qualitative Exploration

**DOI:** 10.7759/cureus.93906

**Published:** 2025-10-05

**Authors:** Nikhilesh Ladha, Naveen Kikkeri Hanumantha Setty, Pankaj Bhardwaj

**Affiliations:** 1 Community Medicine, Employees State Insurance Corporation (ESIC) Medical College, Alwar, Alwar, IND; 2 Community Medicine and Family Medicine, All India Institute of Medical Sciences, Jodhpur, Jodhpur, IND

**Keywords:** india, mdr-tb, patient-centered care, qualitative research, treatment adherence, tuberculosis

## Abstract

Background: Multidrug-resistant tuberculosis (MDR-TB) treatment involves prolonged regimens, multiple adverse effects, and significant psychosocial stressors, often leading to poor adherence. Limited qualitative research from western India has explored patients’ lived experiences during MDR-TB treatment.

Objective: To explore factors influencing treatment adherence among persons with MDR-TB in western India and to identify potential strategies for improving retention in care.

Methods: A qualitative study was conducted in 2018 among 15 patients receiving treatment for MDR-TB at a district-level treatment center in Jodhpur, Rajasthan. Participants were purposively selected to capture variation in age, gender, and place of residence. Data were collected through in-depth, unstructured interviews conducted in the local language (Marwari), audio-recorded with consent, and transcribed verbatim into English. Thematic analysis was undertaken inductively by two independent researchers, followed by consensus coding. Data saturation was considered achieved when no new themes emerged.

Results: Eight key themes emerged: (1) inadequate knowledge and myths about MDR-TB, (2) long pathways to diagnosis and treatment initiation, (3) lack of confidence in treatment efficacy, (4) inability to work and financial hardship, (5) treatment-related side effects, (6) inadequate family and social support, (7) stigma and fear of discrimination, and (8) patient-identified strategies for improvement. Participants suggested shorter regimens, extended drug dispensing intervals, and timely financial support under government schemes as facilitators of adherence.

Conclusions: Treatment adherence among MDR-TB patients in western India is shaped by biomedical, psychosocial, and structural factors. Patient-centered approaches, which address myths, ensure psychosocial and financial support, and introduce flexible, shorter regimens, are crucial for improving treatment outcomes. Strengthening the Direct Benefit Transfer scheme and community-based support programs could play a pivotal role in enhancing adherence and treatment success.

## Introduction

Tuberculosis (TB) is the most common infectious agent-related cause of death worldwide and ranks among the top 10 causes of death. Drug-resistant TB is growing as a public health crisis. Globally, 400000 people are estimated to develop multidrug-resistant TB (MDR-TB) or rifampicin-resistant TB (RR-TB) in 2023. More than half of the world’s cases of MDR/RR-TB are concentrated in five countries: India (27%), the Russian Federation (7.4%), Indonesia (7.4%), China (7.3%), and the Philippines (7.2%). For people who started on treatment in 2021, the treatment success rate of MDR-TB was 68% [1].

Treatment of MDR-TB can be a long process. The latest recommendations by WHO for treating drug-resistant TB include three major categories of regimens. The first category comprises two six-month all-oral regimens for individuals with MDR/RR-TB (with or without resistance to fluoroquinolones). The second category includes several all-oral short regimens of nine months for people with MDR/RR-TB who are not resistant to fluoroquinolones. The third category includes longer regimens of 18-20 months that may consist of an injectable drug (amikacin) [2,3].

If we observe at the national level, according to the India TB report 2024, the incidence of MDR-TB in India is 63,939 cases, with a success rate of only 65% [4]. This low success rate prompts us to examine the factors influencing treatment adherence among MDR-TB patients. While quantitative studies highlight poor MDR-TB treatment outcomes, few qualitative studies in western India have explored patients’ perspectives. This study was conducted during a specific period (i.e., 2018) when the National Program, known as the Revised National Tuberculosis Control Program (RNTCP), was undergoing a transition towards the National Tuberculosis Elimination Program, with a focus on achieving elimination by 2025. Using qualitative methods will help us understand patients’ distinct and unique experiences and the local factors that may help to tailor the program in this area. Although the study is based in a specific region, the findings can be applied to the state and national levels, paving the way for future research in other areas.

## Materials and methods

This qualitative study was conducted in the Jodhpur district of Rajasthan, northwestern India. Rajasthan state is the largest state in India, with 5.7% of India’s population, and contributes 7.1% of MDR-TB cases in India [4]. Jodhpur is one of the largest districts in Rajasthan state, centrally situated in the state's western region, with a population of 3.7 million, according to the 2011 census [5].

This study was part of a thesis for a postgraduate qualification. Participants were selected purposively by the researcher based on the richness of their experience and to ensure variation in age, sex, and rural-urban residence. Eligibility criteria included age ≥18 years, diagnosed with MDR-TB, and currently on treatment. Participants were excluded if they were unable to provide informed consent or if their clinical condition precluded safe and meaningful participation. Sampling continued until thematic saturation was achieved. Ethical clearance was obtained from the Institutional Ethics Committee, All India Institute of Medical Sciences, Jodhpur (Approval No: AIIMS/IEC/2017/292). Written informed consent was taken from all participants. Confidentiality and anonymity were assured by de-identifying transcripts.

A total of 15 participants underwent in-depth interviews, conducted between June and October 2018, using an unstructured interview guide that explored knowledge of TB, treatment experiences, support systems, and barriers to adherence (see Appendix A). The guide was investigator-developed, based on literature, in discussion with experts, pilot-tested, and adapted iteratively. Interviews were conducted in Marwari (local dialect) by the principal investigator, who was fluent in the same, at locations convenient for participants. Each interview lasted 45-60 minutes, was audio-recorded with permission, and supplemented by field notes. The interviewer (principal investigator) was a trained public health professional with prior experience in TB care. Efforts were made to minimize interviewer bias by using open-ended prompts and ensuring participants could speak freely.

Recordings were transcribed verbatim on the same day to prevent information loss and then translated into English. Two researchers independently read and coded transcripts using an inductive thematic analysis approach. Codes were compared and refined through discussion until consensus was achieved. Themes were developed iteratively, supported by illustrative quotes from participants. Coding was conducted manually. The frequencies of the responses were calculated to present the qualitative observations in semi-quantitative form (Table 1).

**Table 1:** Proportion of respondents and qualifiers used

Proportion of respondents	Qualifiers used
<10%	Very few
10-24%	Some
25-49%	Approximately half
50-74%	Most/over half
75-89%	The majority
>=90%	Almost all

Credibility was enhanced through investigator triangulation and repeated readings of transcripts. Transferability was addressed by providing contextual detail about participants and the setting. Dependability was ensured by maintaining an audit trail of coding decisions. Member-checking was not conducted, which is acknowledged as a limitation.

## Results

A total of 15 patients underwent in-depth interviews. The age range was 18 to 65 years. Of the 15 participants, 11 (73.3%) were males, and four (26.7%) were females. Nine (60.0%) participants were from rural backgrounds, and six (40.0%) resided in urban areas. Details of participants are given in Appendix B.

The main themes pertaining to patients’ adherence to treatment that emerged from the data, with supporting quotations, are detailed in Table 2.

**Table 2:** Participants' quotations and themes emerged pertaining to adherence to the treatment MDR-TB: multidrug-resistant tuberculosis

Sr. no.	Themes pertaining to patients’ adherence to treatment	Participants' perspectives in the form of quotations supporting the theme
1.	Inadequate knowledge and myths about MDR-TB	“This (MDR TB) happens because of eating dirty things or because of dust or dirt.. or it occurs from oily (food).” (P 12) “One reason is the tension (Stress), and another is working in mines. That is what is known… Tension is the bigger reason.” (P 14)
2.	Long pathway before treatment initiation	“I did not know about it (TB). One year before, I went to the doctor, where I was told to have TB. I took a complete course from a private (doctor). The course was finished.. then I got a cough and cold again… approximately three months later. So I again went to the hospital.. then an investigation on sputum was done… I did not know.. I thought I was ready (okay) now.., but then I got a call from the hospital.. from our Kamla Nehru (Infectious Disease Hospital for TB). Our Shushila Madam (Treatment supporter) called me and told me that I have MDR TB. Come.. we need to start your treatment.. Then they had many, around 15-20 investigations.. then for starting (treatment), they admitted me for 10 days, and it (treatment) was started.” (P 13)
3.	Lack of faith in treatment efficacy	“When I saw the course, my mind got worse (condition).. because of so many medicines and injections I received, I went into more depression. So, I started taking medication by skipping doses now and then… I felt that these medicines were harming me... It might be possible that they are harming some body parts.” (P 1) “TB might be getting cured or not getting cured.. (I) don’t know but the problem is increasing, and nothing else (happening)” (P 10)
4.	Discontinuation of employment or inability to work	“No one is earning.. I have four daughters.. two sons.. one is young.. one is old and is studying. I had to spoil his studies.. to fill stomach we did labor work.. what else could be done..” (P 3)
5.	Factors related to treatment effect and side effect	“Now see, I had to take injections.. 3 months I got (injections).. 6 more months were remaining. I told them I had severe pain in my buttocks and had to stop injections.” (P 3) “Worsening mind (mental state), losing weight, vomiting, anorexia, no interest in anything.. just lie in bed silently alone.. means this medicine is like this that it makes one sicker like..” (P 1)
6.	Inadequate family support to the TB patient	“It may be someone like me.. half minded.. I also sometimes thought about leaving the treatment.. when I see my in-laws (behavior), I think about leaving (treatment).. finally life will end..” (P 10) “Only a fear remains in mind that I have MDR.. now nothing is there. I also used to feel the same, but due to blessings of doctors means with doctors’ advice.. they made me stronger, and now I feel completely fine from MDR” (P 9, Discussing about the support received from medical team)
7.	Social determinants in the form of discrimination or fear of discrimination	“In the hospital, they do.. stand away… and keep their distance..” (P 4, when asked about discrimination faced) “That my disease won’t spread to others.. I used to do… Used to sit far away.. used to stay away from other household members.” (P 14, while discussing if he faced discrimination and isolated by others)
8.	Ways to improve patient adherence	“Doctors, first of all, need to do this that find a solution for this treatment so that a minimum number of medicines are there.. and medicine should be like this that it should not bother patient and they won’t leave it in between” (P 1, suggesting for shorter treatment regimen) “Only get 4-5 days tablet.. (I) should get it for 10 days.. (I) have to visit thrice a month only.” (P 9, requesting to provide more tablets in one go to decrease frequency of visits and associated cost) “What else.. for medicines.. for petrol to come and go.. what else we can use it for.. For household expenses.. (I) have so much debt..” (P 7, when asked how she utilize the monetary support given by Government of India in form of 500 INR under Nikshay Poshan Yojana to improve nutritional profile of TB patients)

## Discussion

This study highlights the voices and perspectives of patients regarding factors influencing their treatment adherence, offering a rich source of information about the ground realities in MDR-TB care. While the findings from a small qualitative study cannot be generalized, they offer valuable insights into the social, psychological, and structural barriers that influence adherence and may inform improvements in program implementation.

A total of eight major themes emerged. First, most participants had little to no knowledge of the causes and transmission of TB. Misconceptions such as the notion that TB is brought on by stress, diet, or mining activities coexisted with partial knowledge of airborne transmission. Similar findings have been reported elsewhere [6], highlighting the need to enhance communication strategies to dispel myths and promote accurate information about TB.

Second, before arriving at the MDR-TB center, patients recounted a protracted and taxing process that involved numerous consultations and consecutive therapy courses for diagnosis and treatment initiation. Similar incidents in Mumbai [7] were reported, indicating structural deficiencies in the prompt diagnosis and connection of care.

Third, some participants expressed doubt about the efficacy of their current treatment and feared that medications were causing more harm than benefit. Perceived lack of efficacy has been linked with poor outcomes in MDR-TB and other chronic conditions [7-10]. This highlights the importance of consistent counseling and reassurance to sustain confidence in treatment.

Fourth, unemployment and financial distress were nearly universal among participants, often resulting in educational disruptions for family members and exacerbating household poverty. These findings are consistent with previous reports of TB as a “medical poverty trap” [11-13]. Financial hardship is a well-documented determinant of non-adherence [14-16] and underscores the need for stronger social protection measures.

Fifth, treatment side effects, including nausea, vomiting, weakness, and psychological distress, were a major barrier to adherence. Such difficulties have been described in earlier studies in Mumbai and elsewhere [7,17]. Patients often associated side effects with declining health, which reduced trust in medications and increased the risk of treatment interruption.

Sixth, inadequate family support and strained household relationships further weakened adherence. Psychological distress, fear, and hopelessness emerged as recurring experiences. As suggested in prior research [7,18,19], integrating psychosocial counseling and family-centered support into MDR-TB programs may mitigate these challenges.

Seventh, stigma and fear of discrimination were evident both externally and internally. While overt family discrimination was less commonly reported, many participants practiced self-isolation, reflecting internalized stigma. This finding aligns with those from India and other high-burden countries [20,21]. Stigma reduction must remain a top priority for the program.

Eighth, patients themselves identified feasible strategies to improve adherence, including shorter regimens, extended drug dispensing intervals, and timely increased financial assistance. Nutrition consistently emerged as a priority for using Direct Benefit Transfer (DBT) funds. These perspectives are consistent with evidence from South Asia and Africa on the importance of nutritional support [11,22,23]. Flexibility in service delivery and patient-centered approaches have also been linked to better adherence [24-29].

Taken together, these findings illustrate that treatment adherence is not only a biomedical issue but also deeply rooted in the social determinants of health [11,27]. Interventions must therefore extend beyond medications to encompass financial, nutritional, psychosocial, and stigma-related support.

It is worth noticing that the Government of India is constantly working on this pathway. Recent doubling of financial assistance under DBT-Nikshay Poshan Yojna, NikshyaMitra (community support program in India that allows individuals, organizations, and institutions to support TB patients), other new initiatives, including incentivizing treatment supporters and Accredited Social Health Activist (ASHA) workers, TB Vijeta’s (TB champions), and Nikshay SAATHI (family caregiver model), are aimed at enhancing patient support systems further [30]. The introduction of the BPaLM regimen (a four-drug combination - bedaquiline, pretomanid, linezolid, and moxifloxacin) is expected to shorten the traditional MDR treatment, a need that was also voiced in this study.

This study has limitations that could impact the transferability of its findings, including its small sample size, single-district focus, and lack of member checking. Another possibility is that subtleties were lost in the translation from Marwari to English. Nevertheless, the study provides insightful, patient-centered information pertinent to MDR-TB treatment in India.

## Conclusions

This qualitative study shows that complicated biomedical, psychosocial, and structural factors influence treatment adherence in MDR-TB patients in western India. Adherence was hampered by misconceptions regarding the cause of the disease, drawn-out diagnostic processes, financial difficulties, adverse drug reactions, a lack of family support, and stigma. Patients also highlighted the need for more flexible service delivery, shorter regimens, and prompt reimbursement. According to these results, patient-centered, comprehensive approaches that incorporate counseling, psychosocial support, financial and nutritional aid, and more recent, shorter treatment plans are crucial. Strengthening adherence, enhancing treatment results, and advancing India's TB elimination objectives all depend on removing these obstacles.

## Appendices

Appendix A

Semi-structured Interview Guide for Exploring Factors Related to Adherence in MDR-TB Patients

Q1) What do you know about MDR TB? (Probe for Causes, Mode of transmission, Treatment, Prevention)

Q2) How is the treatment affecting you? (Probe for side effects)

Q3) How did you feel when you came to know about the diagnosis of MDR TB? (Probe for coping mechanism)

Q4) How are family & friends treating you? (Probe for isolation and stigma)

Q5) How will you use 500 INR DBT? (Probe for financial insecurities and debt)

Q6) Please provide suggestions that can help the government make treatment better.

Appendix B

**Table 3:** Details of participants

Participant	Age	Gender	Area of residence
P1	34 years	Female	Urban
P2	65 years	Male	Urban
P3	45 years	Female	Rural
P4	65 years	Male	Rural
P5	60 years	Male	Rural
P6	50 years	Male	Rural
P7	40 years	Female	Rural
P8	33 years	Male	Urban
P9	38 years	Male	Rural
P10	26 years	Female	Urban
P11	37 years	Male	Rural
P12	25 years	Male	Urban
P13	18 years	Male	Urban
P14	55 years	Male	Rural
P15	20 years	Male	Rural
